# Endometriosis-Associated Infertility: A Review of Pathophysiological Mechanisms and Current Treatment Strategies

**DOI:** 10.3390/jcm15114297

**Published:** 2026-06-02

**Authors:** Magdalena Gniadek, Alina Porubenska, Karolina Pełka, Zuzanna Rzepkowska, Jerzy Florjański

**Affiliations:** 1 Student Scientific Group of Obstetric and Gynecology, Wroclaw Medical University, 50-368 Wroclaw, Poland; aporubenska13@gmail.com (A.P.); karolina.pelka@student.umw.edu.pl (K.P.); zuzanna.rzepkowska@student.umw.edu.pl (Z.R.); 2 University Centre of Obstetrics and Gynecology, Wroclaw Medical University, 50-556 Wroclaw, Poland; jerzy.florjanski@umw.edu.pl

**Keywords:** endometriosis, ART, IVF/ICSI, infertility, pregnancy, treatment, diagnosis

## Abstract

**Background:** Endometriosis affects 10–15% of reproductive-aged women and is a leading cause of infertility through anatomical, inflammatory, and molecular mechanisms. **Objective:** This review synthesizes current evidence on the pathophysiology of endometriosis-associated infertility and evaluates medical, surgical, and ART strategies to guide individualized management. **Methods:** We conducted a narrative review (2000–2025; PubMed, Scopus, WoS) synthesizing RCTs, meta-analyses, and observational studies on mechanisms and treatment outcomes. **Results:** In rASRM stage III–IV, tubo-ovarian distortion and adhesions mechanically impair oocyte pickup and embryo transport. In superficial disease, animal models demonstrate that peritoneal inflammatory mediators and ROS can impair oocyte maturation, though direct causal evidence in humans is lacking. Epigenetic dysregulation has been identified in the eutopic endometrium and linked to progesterone resistance, though its direct causal role in infertility remains unestablished. Hormonal suppression controls pain but does not improve spontaneous conception rates. Laparoscopic surgery in stage I–II remains debated. Prospective evidence supporting fertility benefit from DIE excision without mechanical obstruction is lacking. Cystectomy consistently reduces AMH, favoring IVF over surgery unless symptoms or retrieval barriers exist. IVF/ICSI live birth rates per cycle in stage I–II are comparable to those without endometriosis; cumulative rates after ≤5 cycles reach 43–46% in treated vs. 28% in untreated stage III–IV patients. **Conclusions:** Management requires two sequential decisions: first, whether to perform a diagnostic laparoscopy to identify minimal disease and adhesions, and second, whether to proceed with surgery or transfer directly to ART. Age ≥ 35, infertility > 2 years, or low AMH/AFC favor immediate IVF. Post-surgical EFI guides timing: high EFI supports expectant management or IUI; low EFI should prompt ART referral. When cystectomy is necessary, tissue-sparing techniques should be prioritized and fertility preservation, including oocyte cryopreservation, discussed preoperatively.

## 1. Introduction

Endometriosis is defined as the presence of endometrial-like tissue outside the uterine cavity, leading to a chronic, estrogen-dependent inflammatory response [1]. It is one of the most common gynecological conditions, affecting approximately 0.5–5% of fertile women and up to 25–40% of those with infertility [1,2]. The clinical course is often heterogeneous and poses a challenge for reproductive medicine.

Endometriosis affects fertility through several overlapping mechanisms. In advanced stages (rASRM stage III–IV), pelvic adhesions and tubal dysfunction mechanically impair oocyte pickup and embryo transport [3,4]. In superficial disease, direct evidence linking peritoneal inflammation to oocyte quality remains limited. Animal models demonstrate that peritoneal ROS can impair oocyte maturation [5], and elevated IL-6 and IL-8 in peritoneal fluid have been shown to exert toxic effects on gametes and embryos [6,7]; however, causal evidence in humans with superficial endometriosis specifically remains lacking. Epigenetic dysregulation has been identified in the eutopic endometrium and linked to progesterone resistance, though its direct causal role in infertility remains unestablished [8,9]. While molecular biomarkers, such as BCL6, are investigated for the non-invasive diagnosis of endometrial inflammation, clinical evidence is currently insufficient to demonstrate their capacity to predict pregnancy outcomes [2,3].

Treatment strategies include medical, surgical, and assisted reproductive technology (ART). Hormonal suppression effectively controls pain but does not improve spontaneous conception rates [10,11]. The effect of laparoscopic surgery on fertility in stage I–II disease remains debated: one systematic review and network meta-analysis reported increased pregnancy rates (OR 1.63; 95% CI 1.13–2.35) [12], while a Cochrane review published in the same year did not confirm this benefit due to insufficient evidence quality [13]. ART—specifically In Vitro Fertilization (IVF) and Intracytoplasmic Sperm Injection (ICSI)—is the main option in cases of advanced disease or tubal involvement [1,3].

Given the variability in disease presentation and reproductive outcomes, treatment should be individualized [2,5]. This review summarizes current evidence on mechanisms and treatment strategies in endometriosis-related infertility.

## 2. Materials and Methods

This study was conducted as a narrative review. A comprehensive literature search was performed using electronic databases, including PubMed, Scopus, and Web of Science. The search covered studies published between 2000 and 2025.

The following keywords and their combinations were used: “endometriosis”, “infertility”, “assisted reproductive technology”, “IVF”, “treatment”, and “pathophysiology”.

Studies were included if they addressed the relationship between endometriosis and infertility or evaluated treatment strategies, including medical, surgical, and ART-based approaches. Priority was given to systematic reviews, meta-analyses, randomized controlled trials, and high-quality observational studies.

Articles not published in English, case reports, and studies lacking relevance to reproductive outcomes were excluded.

The selection process involved screening titles and abstracts, followed by full-text evaluation of eligible studies. The findings were synthesized qualitatively, with emphasis on clinically relevant outcomes and current therapeutic strategies.

## 3. Pathophysiology of Endometriosis in the Context of Fertility

### 3.1. Structural Abnormalities

Structural abnormalities have long been considered the main cause of endometriosis-associated infertility. However, evidence shows that their role extends beyond mechanical obstruction of the reproductive tract. Advanced disease is often associated with ovarian endometriomas, fibrotic adhesions, and distortion of tubo-ovarian anatomy, which may impair ovulation, oocyte pickup, tubal patency, and embryo transport [1,14].

Revised American Society for Reproductive Medicine (rASRM) classification stratifies endometriosis severity based on lesion size, depth, and adhesions [15]. In patients with advanced disease (rASRM stage III–IV), particularly in the presence of significant tubo-ovarian distortion, surgical treatment may be considered to restore pelvic anatomy and potentially improve natural conception, although the benefit is highly dependent on individual factors such as age and ovarian reserve [16]. In contrast, hormonal suppression does not improve fertility outcomes in women with minimal-to-mild disease and should not be used for this purpose. In these patients, ART, including intrauterine insemination or IVF/ICSI, may be considered depending on infertility duration and prognostic factors [16]. Importantly, repeated surgical interventions are generally discouraged due to the lack of clear evidence for improved reproductive outcomes [16]. Furthermore, in patients with ovarian endometriomas, clinicians should carefully balance the potential benefits of surgery against the risk of reduced ovarian reserve [16]. Current evidence indicates that surgery prior to ART does not consistently improve reproductive outcomes and may reduce ovarian reserve, therefore it should not be routinely performed unless clinically indicated (e.g., pain, suspicion of malignancy, or technical issues during oocyte retrieval) [16].

Importantly, the potential benefits of surgical treatment must be balanced against its well-documented impact on ovarian reserve, particularly in patients with ovarian endometriomas. Cystectomy, although associated with lower recurrence rates compared with ablative approaches, has been consistently linked to a decline in ovarian reserve markers, including anti-Müllerian hormone levels, likely due to inadvertent removal of healthy ovarian tissue [17]. Ablative or combined techniques may be less detrimental to ovarian reserve, although their long-term reproductive outcomes remain less clearly defined. Although ablative techniques are quite common, excisional surgery with the stripping of the cyst capsule is the preferred method of endometrioma removal due to lower rate of recurrence compared to the ablation [18], but the choice of surgical method depends on many factors (e.g., size, location of the cyst).

Taken together, the available evidence strongly suggests that surgical endometrioma removal can cause permanent decline in ovarian reserve measured as AMH decline [18].

Overall, integrating anatomical findings with clinical parameters—including patient age, ovarian reserve, disease stage, Endometriosis Fertility Index (EFI), symptom burden, and infertility duration—may support more individualized decision-making and help guide the choice between expectant management, surgery, and ART. A summary clinical decision framework is presented in Table 1.

### 3.2. Systemic Pathophysiological Mechanisms in Endometriosis

Beyond local pelvic distortion, endometriosis also affects immune, metabolic, and oxidative pathways, negatively influencing reproductive function. Unlike acute inflammation, this process remains chronic and self-sustaining, leading to continuous immune activation within the peritoneal cavity, ovaries, and endometrium [23]. Systemic involvement is reflected by altered peritoneal and follicular fluid composition, with increased levels of cytokines, chemokines, prostaglandins, and reactive oxygen species (ROS) [5,6,24]. This inflammatory state is mainly driven by repeated retrograde menstruation and cyclic bleeding of ectopic lesions, which expose the peritoneal cavity to hemolyzed erythrocytes, cellular debris, and danger-associated molecular patterns (DAMPs) [6]. These molecules activate macrophages and stimulate cytokine production, maintaining a chronic inflammatory cycle [6].

At the peritoneal level, adhesion formation and fibrosis are linked to mesothelial-to-mesenchymal transition (MMT), during which mesothelial cells acquire fibroblast-like features [10]. Experimental studies suggest that inflammatory mediators within peritoneal fluid initiate and sustain MMT [10]. IL-33 acting through the ST2 receptor appears to play an important role through activation of TGF-β signaling pathways [7,10]. Activation of the PI3K/AKT/GSK-3β/β-catenin pathway promotes extracellular matrix deposition and cytoskeletal remodeling, facilitating lesion attachment to the peritoneal surface [10]. MMT also disrupts the mesothelial barrier, increasing peritoneal permeability and prolonging exposure of gametes and embryos to inflammatory cytokines, iron, and reactive oxygen species [23,24]. In parallel, ectopic endometrial epithelial cells undergo epithelial–mesenchymal transition (EMT) changes, which enhance survival and resistance to apoptosis [25]. TGF-β1 promotes EMT, myofibroblast differentiation, and collagen synthesis [25]. These mechanisms are further amplified by M2-polarized macrophages, which secrete pro-fibrotic and pro-angiogenic mediators within endometriotic lesions [6,25].

Elevated concentrations of IL-6 and IL-8 in peritoneal fluid have been consistently reported and are thought to sustain leukocyte recruitment, angiogenesis, and lesion survival while simultaneously exerting toxic effects on gametes and embryos [6,7]. These cytokines are also detectable in follicular fluid, where they directly influence granulosa cell function, steroidogenesis, and oocyte maturation [26]. A central mediator linking local and systemic inflammation is monocyte Chemoattractant Protein 1 (MCP-1), which drives monocyte recruitment and macrophage accumulation both within lesions and in the follicular environment [27]. Increased MCP-1 levels in follicular fluid have been associated with intrafollicular inflammation, impaired folliculogenesis, and poorer IVF outcomes, highlighting the direct reproductive consequences of systemic inflammatory spillover [25,27].

Oxidative stress is another important consequence of chronic inflammation. Elevated levels of reactive oxygen species (ROS) have been consistently detected in both peritoneal and follicular fluids of affected women [5,25]. Under these conditions, ROS induce lipid peroxidation, mitochondrial dysfunction, and DNA damage in oocytes, embryos, and endometrial cells, thereby compromising oocyte quality, fertilization potential, and early embryonic development [5]. Beyond direct cellular toxicity, oxidative stress also exerts long-lasting effects through epigenetic reprogramming. Oxidative stress modulates DNA methylation and histone modifications through redox-sensitive pathways and TET enzyme activity [28]. These changes may promote inflammation, progesterone resistance, and lesion survival. As a result, a form of “inflammatory memory” may develop within ectopic and eutopic tissues, reinforcing reproductive dysfunction even in the absence of active bleeding.

Immunologically, endometriosis is increasingly viewed as a condition characterized by a tumor-like immune profile [29]. Macrophages constitute the predominant immune cell population within lesions and peritoneal fluid, where they are skewed toward an M2-like phenotype under the influence of IL-17, IL-10, and TGF-β [29]. Elevated IL-17 levels in peritoneal and follicular fluids link adaptive immune dysregulation directly to ovarian and endometrial dysfunction [29]. IL-17A produced by Th17 cells stimulates secondary cytokines and chemokines, including IL-1β, IL-6, and IL-8 [6]. It also promotes macrophage polarization toward the M2 phenotype. Rather than resolving inflammation, M2 macrophages secrete pro-angiogenic, pro-fibrotic, and immunosuppressive mediators that support lesion survival, fibrosis, and immune evasion [6].

Additionally, dysregulation of uterine natural killer (uNK) cells contributes to systemic immune imbalance [29]. Both excessive cytotoxicity and functional exhaustion of uNK cells have been described, potentially disrupting the delicate immune tolerance required for implantation and early placentation [26]. Emerging evidence further implicates neutrophil extracellular traps (NETs) as contributors to sterile inflammation and tissue damage in endometriosis, reinforcing the chronic inflammatory state [6].

Given the central role of inflammation and oxidative stress in endometriosis, anti-inflammatory drugs and antioxidant supplementation have been investigated as potential adjunct therapies. NSAIDs are widely used for pain relief; however, there is no evidence that they improve fertility outcomes [30]. Similarly, antioxidants (e.g., vitamins C and E, coenzyme Q10, melatonin, N-acetylcysteine) have been studied for their ability to reduce oxidative stress and improve reproductive outcomes. Although some studies report improvement in oxidative stress markers and symptoms, no significant effect on clinical pregnancy rates has been demonstrated, and results remain inconsistent [30,31]. Therefore, these therapies cannot be recommended as standard treatment for infertility in endometriosis.

These data suggest that endometriosis-associated infertility results not only from localized pelvic disease but also from systemic immune and inflammatory dysregulation. Peritoneal and follicular fluids appear to play an important role in linking lesions with impaired reproductive function. Key mechanisms underlying these processes are summarized in Table 2.

### 3.3. Endometriosis and Ovarian Reserve

The relationship between endometriosis and ovarian reserve is complex and remains debated. Ovarian endometriomas were traditionally thought to reduce ovarian reserve through mechanical compression and surgical damage. However, growing evidence suggests that chronic inflammation and oxidative stress may play a greater role in ovarian dysfunction [31]. Histopathological studies of ovarian tissue adjacent to endometriomas show reduced follicular density, stromal fibrosis, increased apoptosis, and premature follicular atresia, even before surgery [27]. Inflammatory infiltration and altered vascularization further suggest active involvement of the ovarian cortex in the disease process [31].

Recurrent bleeding within endometriotic cysts leads to iron accumulation in peritoneal and follicular fluids, promoting reactive oxygen species (ROS) generation and overwhelming antioxidant defenses [32]. Excessive ROS causes mitochondrial dysfunction, lipid peroxidation, and DNA damage in granulosa cells and oocytes, impairing meiotic competence and embryo developmental potential [32].

More recently, iron overload has been linked to ferroptosis, a regulated form of iron-dependent cell death characterized by lipid peroxidation and distinct epigenetic changes. Experimental evidence suggests that ferroptosis may contribute to granulosa cell loss and impaired oocyte quality in endometriosis [32]. These mechanisms provide a plausible explanation for the frequent observation that ovarian reserve markers may remain within normal ranges despite markedly reduced reproductive outcomes.

Nevertheless, conflicting data exist. A recent study reported no significant increase in iron levels or oxidative stress markers in follicular fluid from women with ovarian endometriomas, underscoring disease heterogeneity and methodological variability across studies [5]. Overall, these findings suggest that endometriosis preferentially compromises oocyte quality rather than follicle quantity, with important implications for fertility preservation and ART strategies, but further investigations are necessary. It is still unclear how exactly oxidative stress factors impact the oocyte.

### 3.4. Impaired Endometrial Receptivity to Implantation

Impaired endometrial receptivity is increasingly recognized as a critical determinant of infertility in endometriosis, independent of embryo quality. At the cellular level, dysfunction of decidual stromal cells (DSCs) represents a central pathological feature [24]. Recent studies suggest that aberrant N6-methyladenosine (m6A) RNA methylation suppresses lactate dehydrogenase A (LDHA) expression [32]. This reduces glycolytic flux and ATP production during decidualization. Because decidualization is highly energy-dependent, these metabolic alterations may impair implantation [24].

Moreover, other factors that lead to impaired endometrial receptivity are epigenetic changes. Epigenetic dysregulation extends beyond RNA methylation. Increased H3K27me3-mediated repression of ten-eleven translocation (TET1) genes disrupts DNA demethylation dynamics in the secretory endometrium, resulting in transcriptional rigidity and inadequate activation of implantation-related genes [8]. These epigenetic alterations reinforce progesterone resistance, a well-recognized feature of endometriosis-associated endometrial dysfunction [8].

In parallel, dysregulated miRNA expression profiles further impair endometrial receptivity [33]. Alterations in miR-143-3p, miR-145-3p, miR-99a-5p, miR-31, and miR-1910-3p have been shown to affect cytoskeletal organization, cell adhesion, inflammatory signaling, and progesterone responsiveness in endometrial and decidual cells [34]. Notably, several of these miRNAs are also altered in oocytes and cumulus cells, suggesting coordinated epigenetic dysregulation across multiple reproductive compartments [34].

Thus, implantation failure in endometriosis reflects a convergence of metabolic, epigenetic and post-transcriptional regulatory defects, rather than a single molecular abnormality.

## 4. Diagnosis of Fertility Disorders in Women with Endometriosis

According to the Clinical Practice Guidelines No. 11 of the American College of Obstetricians and Gynecologists, the diagnosis of endometriosis should primarily be based on clinical evaluation, including symptoms and imaging, while laparoscopy should be reserved for selected cases. Current recommendations emphasize a shift toward non-invasive diagnostics and earlier clinical recognition, particularly in women with infertility.

Endometriosis significantly affects fertility and is often diagnosed several years after the onset of symptoms [16]. Delayed diagnosis may lead to disease progression, formation of adhesions, and increased severity of pelvic lesions, all of which impair reproductive potential [35].

Although clinical symptoms alone are not sufficient for diagnosis, their assessment provides important information about disease location and extent. Clinical examination remains the first diagnostic step; however, its sensitivity is limited, particularly for deep infiltrating lesions [36]. A comprehensive gynecological examination, including bimanual and rectovaginal assessment, is recommended [16]. Reduced uterine mobility is considered a clinical predictor of endometriosis-associated infertility, reflecting pelvic adhesions and anatomical distortion [37].

Endometriosis is classified into superficial peritoneal lesions, ovarian endometriomas, and deep infiltrating disease [16]. Imaging plays a key role in diagnosis, with transvaginal ultrasound as the first-line modality, especially in women planning pregnancy. Both ultrasound and MRI are effective in detecting ovarian and deep lesions but have limited value in identifying superficial disease [16,38]. Notably, negative imaging does not exclude endometriosis.

Laparoscopy, previously regarded as the diagnostic gold standard, is now reserved for selected cases, including inconclusive non-invasive evaluation, lack of response to empirical treatment, or unexplained infertility [36]. This approach reduces diagnostic delay while limiting unnecessary surgical intervention.

## 5. Current Methods of Treatment of Endometriosis in Women Trying to Conceive

Management of endometriosis should be individualized, taking into account age, ovarian reserve, duration of infertility, coexisting infertility factors, disease stage, prior surgery, and the pain severity [39,40]. Treatment decisions are commonly based on the rASRM classification; however, its value in predicting fertility outcomes is limited [16,41]. The Endometriosis Fertility Index (EFI) provides a more accurate estimation of spontaneous pregnancy after surgery and supports decision-making regarding the timing of assisted reproductive technologies (ART) [21,41,42]. Treatment includes pharmacological, surgical, and assisted reproductive approaches [35].

### 5.1. Role of Ovarian Reserve Markers in Treatment Selection

Assessment of ovarian reserve using anti-Müllerian hormone (AMH) and antral follicle count (AFC) is essential for clinical decision-making in women with endometriosis. These markers are key predictors of ovarian response and reproductive potential and should guide the choice between expectant management, surgical intervention, and early referral to assisted reproductive technologies (ART) [16,43]. Lower AMH and AFC values are associated with diminished ovarian reserve and poorer ART outcomes, and in such cases, repeated surgical procedures should be avoided in favor of timely fertility treatment [14,43]. Conversely, in women with preserved ovarian reserve, more conservative approaches may be considered, particularly in early-stage disease. Preoperative assessment of AMH and AFC is especially important in patients with endometriomas, as it helps balance the potential benefits of surgery against the risk of iatrogenic ovarian damage [16].

### 5.2. Pharmacological Methods

Analgesics may relieve dysmenorrhea but are ineffective for non-menstrual pain [35]. Nonsteroidal anti-inflammatory drugs (NSAIDs) may inhibit ovulation and should be used only short-term [44]. Gonadotropin-releasing hormone (GnRH) agonists and antagonists can be used short-term in selected patients, particularly before ART or surgery [45]. Long-term use is limited by hypoestrogenism and bone loss; add-back therapy is required [46]. Aromatase inhibitors (letrozole, anastrozole) are reserved for refractory cases. In women planning pregnancy, they may be used briefly for ovulation induction, but are not routinely recommended due to adverse effects and limited long-term data [16,47]. According to ESHRE guidelines, hormonal therapy is effective for symptom control but does not improve fertility outcomes [40]. Progestogens effectively reduce symptoms and may be used before planned pregnancy. Ovulation typically resumes after discontinuation. Danazol and antiprogestogens are not recommended due to adverse effects [48]. Postoperative hormonal therapy may reduce recurrence but should be individualized in women planning pregnancy.

### 5.3. Surgical Methods

Surgical treatment plays an important role in women with endometriosis who are planning pregnancy, particularly when conservative management is ineffective or when extensive lesions and adhesions impair fertility [16,49]. Surgery is mainly indicated in cases of anatomical abnormalities affecting tubal function, ovulation, or embryo implantation, as well as in deep infiltrating endometriosis involving structures such as the bladder, intestines, or peri-tubal tissues [16].

Laparoscopy is preferred over open surgery due to reduced postoperative pain, shorter hospitalization, and faster recovery [16]. Evidence suggests that operative laparoscopy may improve spontaneous conception rates in women with minimal or mild endometriosis (rASRM I–II), although its fertility benefits in advanced disease remain uncertain [16,39]. Therefore, surgical decisions should be individualized and based primarily on symptom severity and disease extent. Importantly, surgery may improve quality of life, sexual function, and pain control, indirectly supporting infertility treatment outcomes [16]. However, surgical intervention is not recommended solely to enhance fertility in asymptomatic patients.

### 5.4. Non-Medical Treatments

Non-medical approaches may improve quality of life in women with endometriosis, although they do not constitute a direct treatment for infertility [16]. These strategies do not interfere with ovulation or delay conception attempts and may help reduce pain and improve adherence to treatments such as surgery or ART [50]. Lifestyle interventions, including regular physical activity and balanced nutrition, are recommended as supportive measures for women attempting to conceive. Nevertheless, current evidence does not confirm a direct beneficial effect of these interventions on fertility outcomes [16].

### 5.5. Assisted Reproductive Technologies

Assisted reproductive technologies are an important component of fertility management in women with endometriosis [51]. Intrauterine insemination (IUI), usually combined with controlled ovarian stimulation, may be considered in women with rASRM stage I–II disease and preserved tubal patency [16]. In selected patients, IUI is often used before proceeding to more advanced ART methods [52]. The effectiveness of ART in women with minimal or mild endometriosis is generally comparable to outcomes in women without the disease. However, in advanced stages (rASRM stage III–IV), lower implantation and clinical pregnancy rates, as well as potentially reduced live birth rates, have been reported [16,39]. Endometriosis may also negatively affect ovarian response to stimulation and fertilization outcomes, particularly in severe disease, although no optimal stimulation protocol has yet been established for this patient group [16,39].

## 6. Evaluation of the Effectiveness of Methods of Treating Pain and Infertility in Women with Endometriosis

### 6.1. Pharmacological Methods

According to the 2022 ESHRE (European Society of Human Reproduction and Embryology) guidelines, the recommended treatment for patients with endometriosis-related pain is pharmacotherapy using combined hormonal contraceptives (CHC), progestogens, GnRH (Gonadotropin-Releasing Hormone) agonists, or GnRH antagonists [16]. Based on NICE 2017 (National Institute for Health and Care Excellence), all of the above-mentioned therapeutic options are associated with a reduction in pain compared to groups of patients using placebo [43]. Hormonal therapy has not been shown to negatively affect disease progression, and its side effects are limited; therefore, its use is strongly recommended [53].

Combined hormonal contraception has a beneficial effect on menstrual pain, dyspareunia, and non-menstrual pain [54,55]. Continuous therapy is recommended over standard cyclic therapy. It can result in hormonal balance, which increases the effectiveness of therapy [56]. Available evidence remains insufficient comparing different methods of use oral contraceptives (OCs) (vaginal contraceptive ring, transdermal patch), so it is not possible to select the best one [57].

A study by de Souza Gaio G. et al. (2025) demonstrated similar efficacy of COCP (combined oral contraceptive pills) and progestogens in pain relief, as well as a similar profile of adverse effects, therefore both methods are considered equally effective [58]. Other studies also indicate similar efficacy of these methods [59,60,61].

In a meta-analysis by Muzii L. et al. (2023), nine studies were analyzed to assess the effect of dienogest on reducing pain associated with endometriosis. Its efficacy in pain relief was proven to be comparable to other methods; however, it was more frequently associated with the occurrence of adverse effects such as weight gain, compared to GnRH [62].

Lan S. et al. (2013) analyzed 5 RCT’s (Randomized Controlled Trial) studies, which showed similar efficacy of levonorgestrel-releasing intrauterine systems and GnRH agonists [63]. A study by Jeng CJ. et al. (2014) compared the treatment outcomes of OCs and progestogens vs. GnRH agonists [64]. Due to the more serious side effects of GnRH agonists, progestogens or OCs are considered to be the first-line drugs [63,64,65].

Brown J. et al. (2010) analyzed various routes of administration of GnRH agonists and showed that intramuscular, subcutaneous, or intranasal administration do not differ in their effectiveness [66]. GnRH antagonists appear to be a better method compared to GnRH agonists due to the lack of exacerbation of symptoms at the beginning of treatment [67]. Nevertheless, both agonists and antagonists have similar treatment outcomes [67]. Due to the side effect of hypoestrogenism, both GnRH antagonists and agonists should not be used in adolescents (young women may not yet have reached their maximum bone density) [62,68,69]. Combination therapy with low-dose estrogens or combined oral contraceptives (COCs) may reduce hypoestrogenic adverse effects without compromising pain control [68,70].

A study by Dick A. et al. (2025) comparing the impact of GnRH antagonists and synthetic progestogens showed that GnRH antagonists provide greater benefit in relieving menstrual pain, while progestogens are more effective in treating dyspareunia, confirming the need to tailor treatment to the individual needs of each patient [71]. For young patients, OCs (oral contraceptives) are usually recommended due to the beneficial effect of estrogens on quality of life and the frequent need for contraception in these patients [65]. In the case of combined hormone therapy, those containing low doses of estrogens should be chosen [72,73,74]. Progesterone-only therapies were previously recommended mainly for selected patients, including smokers, women over 35 years of age, and patients at increased thrombotic risk [75,76].

According to guidelines, drugs that inhibit ovarian function (danazol, progestogen, OCP, GnRH agonists) should not be used to improve fertility in infertile patients with endometriosis, as there is no evidence of their effectiveness [76]. At the same time, previous reviews have analysed the effect of GnRH agonists on fertility when combined with other methods of ovulation suppression. In the study by Ibrahim Alkatout et al. (2013), although the evidence is not of high quality, a beneficial effect on fertility (comparable to surgical laparoscopy) is suggested for the GnRH agonist alone [77]. Therefore, further research is needed to determine the effectiveness of this approach.

### 6.2. Surgical Methods

Surgical treatment may relieve pain associated with endometriosis and improve sexual functioning and overall quality of life [78,79,80]. Laparoscopy is preferred over open surgery because of shorter hospitalization and faster recovery. Moreover, laparoscopic excision of lesions appears more effective than ablation in reducing endometriosis-related and chronic pelvic pain [81].

In women with ovarian endometriomas ≥ 3 cm, cystectomy is generally considered superior to drainage and coagulation [82]. Comparable outcomes have also been reported with CO_2_ laser techniques [83,84,85]. However, surgical treatment may negatively affect ovarian reserve; therefore, ovarian reserve assessment and the risk of iatrogenic ovarian damage should be carefully evaluated before surgery, particularly in patients desiring pregnancy [86,87].

For patients with deep infiltrating endometriosis (DIE), surgery may improve pain symptoms [81,88], although it carries a risk of significant complications [89]. Consequently, patients should be thoroughly counseled regarding potential benefits and risks, and procedures should preferably be performed in specialized centers [90]. Surgery is mainly indicated in cases of bowel or ureteral obstruction or failure of medical treatment [91]. Prospective evidence supporting improved fertility after surgical excision of DIE in the absence of mechanical or tubal obstruction is lacking [16].

Reproductive plans should be considered during treatment selection. Fertility preservation strategies, including oocyte vitrification or ovarian tissue cryopreservation, may be offered to women planning pregnancy [92,93]. In women not seeking conception, postoperative hormonal therapy may reduce recurrence risk and improve long-term symptom control [94,95,96].

In bowel endometriosis, radical excision of lesions is generally recommended [16]. Segmental resection should be considered for sigmoid involvement, whereas treatment of rectal DIE requires an individualized approach [16]. Discoid excision has been associated with shorter hospitalization and a lower risk of postoperative bowel stenosis compared with segmental resection [97]. Current guidelines recommend discoid resection for isolated rectal lesions < 3 cm, while segmental resection is preferred in more extensive disease [16,98]. Overall, the preferred strategy is complete but minimally traumatic excision of lesions.

In women with concomitant adenomyosis who do not desire future pregnancy, hysterectomy may be considered [99]. Although favorable long-term outcomes have been reported [100], hysterectomy remains a radical procedure associated with infertility, early menopause, and the possibility of persistent pain due to residual disease [101,102]. Therefore, total hysterectomy is generally preferred over subtotal hysterectomy [16].

Because of the heterogeneity of disease presentation and surgical techniques, no single operative approach can be universally recommended for DIE [16]. Treatment decisions should therefore be individualized. In infertile women with endometriosis, laparoscopic surgery may increase pregnancy rates [12,13,103]. A systematic review by Ruth Mary Hodgson et al. (2020) reported a higher possibility (odds ratio = 1.63; 95% confidence interval, 1.13–2.35) of conceiving after laparoscopic surgery compared to the placebo group [12]. Nevertheless, evidence remains limited, and current recommendations are relatively weak because of the lack of studies directly comparing spontaneous conception rates with and without prior surgical intervention [83,104].

### 6.3. Assisted Reproductive Technology (ART)

Some infertile patients with endometriosis may benefit from ART [13]. The group that may potentially benefit should be identified based on the EFI [16,41]. Infertile patients with stage I/II endometriosis (rASRM) may benefit from IUI after ovarian stimulation. However, obtaining a sufficient number of oocytes may remain a difficulty, despite intensive ovarian stimulation. In a study by Zimmermann A. et al. (2023), a significantly lower number of oocytes was retrieved from women with endometriosis compared to the control group (women without endometriosis), despite the use of high doses of gonadotropins [105]. In contrast, a study conducted by Werbrouck E. et al. (2006) showed that patients with mild or moderate endometriosis who underwent IUI with ovarian stimulation 6 months after surgical treatment achieved similar results to patients with unexplained infertility [106].

ART is particularly beneficial in cases of coexisting male-factor infertility or tubal dysfunction [41,107]. Current evidence indicates that ART is safe in women with endometriosis and does not increase the risk of symptom recurrence [107,108]. Therefore, ART should be considered in patients who fail to achieve pregnancy using other treatment strategies.

In advanced disease (rASRM stage III/IV), implantation potential and live birth rates may be reduced. Tew M.P. et al. (2024) showed that moderate-to-severe endometriosis and concomitant adenomyosis were associated with poorer cumulative IVF/ICSI live birth rate prognosis [109]. Nevertheless, reproductive outcomes appear more favorable in treated versus untreated patients. Zhong Ch. et al. (2021) demonstrated higher cumulative live birth rates (CLBR) in women receiving treatment (up to 5 IVF/ICSI cycles) compared with untreated women (43.6% and 46.3% vs. 27.7%, respectively) [110]. In a study by Sara Alson et al. (2025), after three cycles of IVF/ICSI, women with endometriosis or adenomyosis had lower CLBRs than healthy women (52.2%/70% vs. 68.7%/85.7%, respectively, depending on the test used) [111].

In patients who have undergone cystectomy, there is a risk of reduced ovarian reserve. This is supported by a systematic review conducted by Francesco Raffi et al. (2012), which analyzed 237 patients and found a significant decrease in AMH levels following cystectomy [17]. This may result from the accidental removal of a larger amount of ovarian tissue. When comparing cystectomy with ablation using CO2 laser technology, it appears that the second technique may be less destructive. However, this evidence should be interpreted with caution, as the authors themselves note that it is not possible to draw definitive conclusions [112]. There are studies suggesting potential fertility benefits of embryo cryopreservation in women with endometrioma who undergo cystectomy. In a retrospective study involving 17 patients who underwent embryo freezing prior to cystectomy, it was demonstrated that the miscarriage rate was significantly lower (0% vs. 35.5%) than in the group of women whose embryo transfer was not preceded by this procedure [113]. Although there is evidence supporting a higher likelihood of spontaneous pregnancy following laparoscopic cystectomy, the adverse effect on ovarian reserve confirmed by studies causes that current guidelines recommend performing these procedures only in cases of larger cysts or symptoms that do not respond to medical treatment [108]. Future studies should focus on elucidating the mechanisms leading to a reduction in ovarian reserve, with the aim of developing surgical techniques that combine thorough resection of endometriosis lesions with maximum preservation of normal ovarian tissue [114].

A protocol using GnRH agonists may be more beneficial for patients after cystectomy, but there is no conclusive evidence [115]. In a study by Apostolos Kaponis et al. (2019), a group of women who underwent 3 months of GnRH agonist therapy was compared with a group that did not receive this treatment. A higher ovulation rate was observed in the first group [116]. However, prolonged exposure to GnRH agonists can cause pituitary desensitization, resulting in the need for higher doses of gonadotropins to stimulate ovulation. At the same time, studies show that the short and ultra-long regimens do not affect pregnancy outcomes following ART [117].

There is currently no evidence to suggest that any particular stimulation protocol prior to IVF is superior to others (long GnRH agonist protocols vs. short GnRH antagonist protocols) [118]. The ovarian stimulation index and pregnancy outcomes were also compared between groups using the progesterone-primed ovarian stimulation (PPOS) protocol and the GnRH agonist protocol. Similar results were obtained in both groups [119]. In summary, the current scientific evidence does not point to a specific treatment pathway.

## 7. Limitations

Several limitations of this review warrant consideration. First, as a narrative review, this work is inherently subject to selection bias, as it does not employ the exhaustive searching and formal screening protocols typical of a systematic review. Second, the synthesis of data is challenged by the significant heterogeneity across clinical studies, particularly concerning the inconsistent staging of endometriosis (e.g., rASRM vs. ENZIAN) and the diverse clinical profiles of infertile populations, ranging from minimal peritoneal lesions to complex deep infiltrating disease. Furthermore, the absence of a formal quality assessment (risk of bias analysis) for the cited literature means that the evidence levels vary. Consequently, our findings should be interpreted with caution, emphasizing the urgent need for standardized, large-scale prospective trials to establish more definitive clinical guidelines for managing endometriosis-associated infertility.

## 8. Conclusions

Endometriosis-associated infertility results from the convergence of anatomical distortion, systemic inflammation, and impaired endometrial receptivity. Effective management requires sequential, individualized decision-making rather than a standardized protocol.

### 8.1. Clinical Decision Framework

Establish diagnosis and disease extent. Transvaginal ultrasound is the first-line modality, effective for ovarian and deep infiltrating lesions, but has limited sensitivity for superficial peritoneal disease; negative imaging does not exclude endometriosis. Laparoscopy remains the only method to identify superficial lesions and should be reserved for inconclusive non-invasive evaluation, failed empirical treatment, or unexplained infertility.

Assess ovarian reserve before any intervention. AMH and AFC must be measured before treatment, particularly before surgery. Low values should prompt early ART referral and preclude further surgical procedures, especially in patients with endometriomas, where cystectomy carries a well-documented risk of iatrogenic AMH decline.

Select treatment based on age, reserve, stage, and symptoms. Hormonal suppression effectively controls pain but does not improve spontaneous conception rates. Laparoscopic surgery in rASRM stage I–II remains debated: a network meta-analysis supports improved pregnancy rates, while a contemporaneous Cochrane review did not confirm this benefit. Surgery should not be performed solely to enhance fertility in asymptomatic patients. DIE excision without mechanical obstruction has no prospective evidence of fertility benefit. When cystectomy is necessary, tissue-sparing techniques should be prioritized.

Apply EFI post-surgically. High EFI supports expectant management or IUI; low EFI should prompt ART referral.

Proceed to ART when indicated. IVF/ICSI is the preferred strategy for advanced disease, diminished reserve, age ≥ 35, infertility >2 years, or failure of prior treatment. Outcomes in stage I–II are comparable to women without endometriosis; cumulative live birth rates after up to five cycles reach 43–46% in treated versus 28% in untreated stage III–IV patients. Fertility preservation, including oocyte or embryo cryopreservation, should be discussed preoperatively in patients facing cystectomy or progressive disease.

### 8.2. Final Message

Avoiding unnecessary surgery, minimizing cumulative ovarian damage, and prioritizing timely ART referral are the cornerstones of managing endometriosis-associated infertility. Future research should focus on non-invasive biomarkers predicting treatment response and on large-scale RCTs clarifying the fertility impact of surgery in early-stage disease.

## Figures and Tables

**Table 1:** Clinical decision framework in endometriosis-associated infertility.

Patient Profile	Key Factors	Recommended Approach	Rationale	Key References
Young (<35), good ovarian reserve, infertility, minimal–mild disease (rASRM stage I–II)	Regular cycles, no major anatomical distortion, no other infertility factors	Expectant management or IUI with potential extension to IVF	Limited anatomical disruption; avoid overtreatment	[19]
Moderate–severe disease (rASRM stage III–IV), distorted tubo-ovarian anatomy	Adhesions, impaired tubo-ovarian relationship	Consider surgery vs. IVF	Surgery may restore anatomy but outcomes vary	[20]
Favourable prognosis (high EFI score)	Preserved ovarian function	Surgery + natural conception	EFI predicts pregnancy	[21]
Low ovarian reserve/age ≥ 35	Low AMH/AFC	IVF	Time-sensitive fertility decline	[10]
Endometrioma present	Ovarian involvement	IVF preferred unless symptoms	Surgery may reduce ovarian reserve	[22]
Previous ovarian surgery	Risk of diminished reserve	Avoid repeat surgery	Cumulative ovarian damage	[17]
Multiple failed treatments	Prior failures	IVF	Escalation strategy	[10]

**Table 2:** Key mechanisms of endometriosis-associated infertility.

Mechanism	Key Mediators	Impact on Fertility	Evidence Level	Key References
Inflammation	IL-1β, IL-6, TNF-α, IL-33, IL-17A, Th17 cells, NETs, DAMPs, uterine NK cells (uNK)	Impaired fertilization and implantation, toxic pelvic microenvironment, reduced oocyte competence	Moderate	[5,6,17,26,29]
Adhesion formation and fibrosis	TGF-β, mesothelial-to-mesenchymal transition (MMT)	Tubo-ovarian distortion and impaired gamete transport	High	[10]
Oxidative stress	Reactive oxygen species (ROS)	Oocyte mitochondrial damage; embryo fragmentation	Moderate	[5,25]
Iron/ferritin/ heme	Iron-loaded erythrocyte remnants, siderophages	Fenton reaction; ferroptosis; oxidative stress amplification	Moderate	[28,32]
EMT and enhanced cell survival (epithelial to mesenchymal transition)	TGF-β, Wnt/β-catenin	Persistence of ectopic lesions	Experimental	[7]
Epigenetic mechanisms	DNA hypermethylation of progesterone-responsive genes, H3K27me3-mediated repression of TET1	Inflammatory gene expression and failure to resolve inflammation	Experimental	[8]
miR dysregulations	miR-143-3p upregulation, miR-99a-5p downregulation, miR-31 dysregulation, miR-1910-3p overexpression	Pro-inflammatory stromal phenotype, intrafollicular inflammation, amplified cytokine production	Experimental	[29,30]

## Data Availability

No new data were created or analyzed in this study. Data sharing is not applicable to this article.

## Abbreviations

The following abbreviations are used in this manuscript:

ART	Assisted reproductive technologies
IVF	In Vitro Fertilization
ICSI	Intracytoplasmic sperm injection
rASRM	Revised American Society for Reproductive Medicine
EFI	Endometriosis Fertility Index
GnRH	Gonadotropin-releasing hormone
ROS	Reactive oxygen species
NSAIDs	Nonsteroidal anti-inflammatory drugs
uNK	Uterine natural killer
EMT	Epithelial–mesenchymal transition
MMT	Mesothelial-to-mesenchymal transition
CLBR	Cumulative live birth rates
DIE	Deep infiltrating endometriosis
IUI	Intrauterine insemination
COC	combined oral contraceptives
OCs	Oral contraceptives
CHC	Hormonal contraceptives
COCP	Combined oral contraceptive pills
